# Structure of the native Sec61 protein-conducting channel

**DOI:** 10.1038/ncomms9403

**Published:** 2015-09-28

**Authors:** Stefan Pfeffer, Laura Burbaum, Pia Unverdorben, Markus Pech, Yuxiang Chen, Richard Zimmermann, Roland Beckmann, Friedrich Förster

**Affiliations:** 1 Department of Molecular Structural Biology, Max-Planck Institute of Biochemistry, Am Klopferspitz 18, D-82152 Martinsried, Germany; 2 Department of Biochemistry, Gene Center and Center for integrated Protein Science Munich, University of Munich, Munich D-81377, Germany; 3 Department of Medical Biochemistry and Molecular Biology, Saarland University, Homburg D-66421, Germany

## Abstract

In mammalian cells, secretory and membrane proteins are translocated across or inserted into the endoplasmic reticulum (ER) membrane by the universally conserved protein-conducting channel Sec61, which has been structurally studied in isolated, detergent-solubilized states. Here we structurally and functionally characterize native, non-solubilized ribosome-Sec61 complexes on rough ER vesicles using cryo-electron tomography and ribosome profiling. Surprisingly, the 9-Å resolution subtomogram average reveals Sec61 in a laterally open conformation, even though the channel is not in the process of inserting membrane proteins into the lipid bilayer. In contrast to recent mechanistic models for polypeptide translocation and insertion, our results indicate that the laterally open conformation of Sec61 is the only conformation present in the ribosome-bound translocon complex, independent of its functional state. Consistent with earlier functional studies, our structure suggests that the ribosome alone, even without a nascent chain, is sufficient for lateral opening of Sec61 in a lipid environment.

## Abstract (web summary)

The protein-conducting channel Sec61 is responsible for protein transport and membrane insertion at the endoplasmic reticulum. Here, the authors determine the structure of ribosome-bound Sec61 in a native context, in which it adopts a laterally open conformation, irrespective of its functional state.

## Graphical abstract

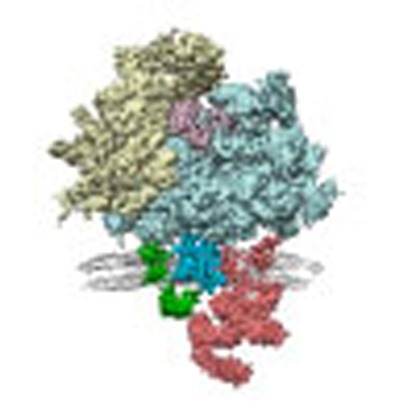

In eukaryotic cells, many proteins have to be translocated across or inserted into the endoplasmic reticulum (ER) membrane during their synthesis^1^. These processes are facilitated by the translocon, a multi-subunit complex located in the ER membrane. The universally conserved heterotrimeric protein-conducting channel Sec61 forms the core of the translocon and binds to translating ribosomes for co-translational protein transport. The structure of the prokaryotic Sec61 homologue, the SecY complex, has been extensively studied by X-ray crystallography^2^,^3^,^4^. Single particle cryo-electron microscopy (EM) studies have provided high-resolution structures of solubilized and purified ribosome-Sec61/SecYEG complexes in defined functional states^5^,^6^,^7^. On the basis of these structures, a detailed model for protein translocation through a central pore and protein membrane insertion via a lateral gate, formed by two adjacent transmembrane helices (TMHs) of Sec61α, has been proposed^2^,^6^,^7^. Specifically, two major conformations are thought to underlie the two fundamentally different functional states of Sec61: (I) in the ‘non-inserting state' (idle or translocating a soluble polypeptide chain), Sec61 is believed to adopt a compact conformation with a closed lateral gate (‘closed' conformation); (II) in the ‘inserting state' (integration of a TMH into the membrane), Sec61 is supposed to transiently adopt a less compact conformation with an open lateral gate (‘open' conformation). Recently, structures of the solubilized ribosome-Sec61 complex purified from porcine pancreas were determined for the two non-inserting conditions, revealing the binding moieties of Sec61 at unprecedented resolution (3.4 and 3.9 Å, respectively)^8^. To date all structural studies that resolved conformations of Sec61 focused on the detergent-solubilized protein-conducting channel in the absence of all other translocon components. Thus, the structure of Sec61 in its native context remains uncharted territory.

Cryo-electron tomography (CET) in combination with subtomogram analysis is an excellent method for studying the structures of large macromolecules in their natural environment^9^,^10^. It is particularly attractive for studying membrane-embedded and -associated complexes, because detergent solubilization is not required, avoiding destabilization of the complex and possible misinterpretation of the density of the detergent micelle surrounding a solubilized membrane protein. CET provided first insights into the structure of the native translocon^11^ and further studies based on isolated rough ER (rER) vesicles from canine and human origin allowed identification of Sec61, the translocon associated protein complex (TRAP) and the oligosaccharyl-transferase (OST) complex in a map of the native translocon at 20 Å resolution^12^. By classification approaches, the latter study also revealed compositional heterogeneity of the translocon; while TRAP is a stoichiometric component of the ribosome-bound translocon complex, OST was found to be present in only 40–70% of translocon complexes. Subnanometer resolution subtomogram averages of membrane protein complexes have recently been obtained from ∼100,000 asymmetric units, which can be collected relatively efficiently for symmetrical samples such as virus coats using automated CET data acquisition^13^,^14^,^15^. Recent revolutionary developments in direct detector technology^16^ suggest that subnanometer resolution subtomogram averages are in reach for much smaller data sets and hence for non-symmetrical macromolecules.

Benefitting from these developments in direct detector technology, we obtained a subtomogram average of mainly idle mammalian ribosomes bound to the native translocon in rER vesicles at subnanometer resolution. This structure allows determining the conformation of native Sec61 in a non-inserting state, revealing an open lateral gate even in the absence of a nascent transmembrane helix, breaking with the current dogma of ER translocation.

## Results

### Structure determination

For structure determination of the ribosome-bound translocon in its native membrane environment, we applied CET and subtomogram analysis to rER vesicles isolated from canine pancreas. Functional assays and western blotting have shown that these rER vesicles contain all protein components necessary for co-translational protein transport and membrane protein integration, as well as subsequent maturation steps of nascent peptides^11^,^12^. In our tomograms, rER vesicles have a diameter of 100–300 nm and are densely populated with membrane-bound ribosomes (Supplementary Fig. 1). From 211 single-axis tomograms recorded on a Titan Krios (FEI) equipped with energy filter and K2 Summit direct detector (both Gatan), we retrieved 105,500 candidate particles by a six-dimensional cross-correlation search^17^ with a ribosome template (‘template matching'). In a first round of classification, we separated false positives and the majority of non membrane-bound ribosomes from ER-associated ribosomes (27,500 remaining subtomograms), which were subsequently subjected to iterative subtomogram alignment. In a second round of classification, ribosomes bound to the OST-containing translocon were separated from remaining non membrane-bound ribosomes and ribosomes bound to the OST-lacking translocon (Supplementary Fig. 2). Systematic screening for optimal tilt series acquisition parameters showed a deteriorating influence of high tilt images on the overall quality of the subtomogram average, likely resulting from specimen alteration due to accumulating beam damage, low signal-to-noise ratio and the inferior alignment of high tilt images into the common three-dimensional coordinate system of the tomogram. To overcome these deteriorating effects and to restrict the cumulative electron dose to ∼30 e Å^−2^, the remaining 17,600 subtomograms were reconstructed from projections covering only a strongly reduced tilt range (−20° to 20°).

### Overall structure of the native ER translocon

**Figure 1:**
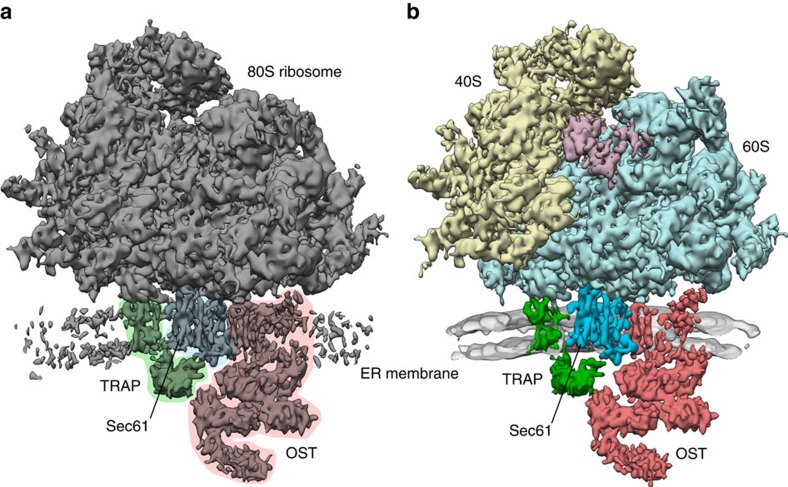
Overall structure of the ER membrane-associated mammalian ribosome. (**a**) Subtomogram average of the ER membrane-associated ribosome filtered to 9.0 Å resolution. In the otherwise unprocessed map, TMHs for Sec61 (blue), TRAP (green) and OST (red) can be distinguished clearly in the membrane. The membrane part of the average has been cut to better visualize the membrane integral parts of the translocon. (**b**) Segmented densities for the 40S (yellow) and 60S (light blue) ribosomal subunits, translation elongation factors (magenta), Sec61 (blue), TRAP (green) and OST (red). Density for the ER membrane (grey) has been filtered to 2 nm resolution and was cut to emphasize the membrane integral parts of the translocon.

After refinement of the subtomogram alignment, the segment of the resulting subtomogram average (Fig. 1) that represents the ribosome is resolved to ∼9 Å, as estimated by Fourier cross-resolution and Fourier shell correlation (FSC) of averages from two halves of the data (Supplementary Fig. 3a). Distinguishable features, such as rod-like densities co-localizing with α-helices in a superposed atomic model of the ribosome (Supplementary Fig. 3b) are consistent with the estimated resolution. In the translocon part of the subtomogram average, we clearly distinguish TMHs for Sec61, TRAP and OST (Fig. 1). Notably, the density accounts for only one copy of Sec61 in addition to TRAP and OST and also extensive classification of subtomograms focused on the ribosome-bound translocon does not yield densities that might account for oligomeric states of Sec61. For most of the ribosome-associated membrane part of the average, the local resolution is determined to be better than 10 Å (Supplementary Fig. 3c). In contrast, local resolution estimation suggests that the lumenal parts of TRAP and OST are resolved to only 12–15 Å. In line with this observation, classification focused on the translocon part of the average revealed that ribosome binding to the translocon is not entirely homogeneous, but allows for minor tilting of the ribosome (up to ∼5° deviation from the average orientation), along an axis formed by its membrane contacts (Supplementary Fig. 4). While this tilting barely affects directly ribosome-associated translocon density, misalignment of the translocon part increases into the lumen, resulting in worse resolution for the lumenal parts of TRAP and OST. Inherent flexibility of TRAP and OST might contribute to the systematically deteriorating resolution towards the ER lumen. Subtomogram alignment focused on the lumenal parts of the translocon did not yield satisfactory results, probably because the signal of subtomograms excluding the ribosome was insufficient for accurate alignment. As a control for the ‘conventional' alignment procedure, we performed subtomogram alignment following the ‘gold standard' procedure, which yielded essentially identical results (Supplementary Fig. 3a).

### Visualized Sec61 complexes are in the non-inserting state

**Figure 2:**
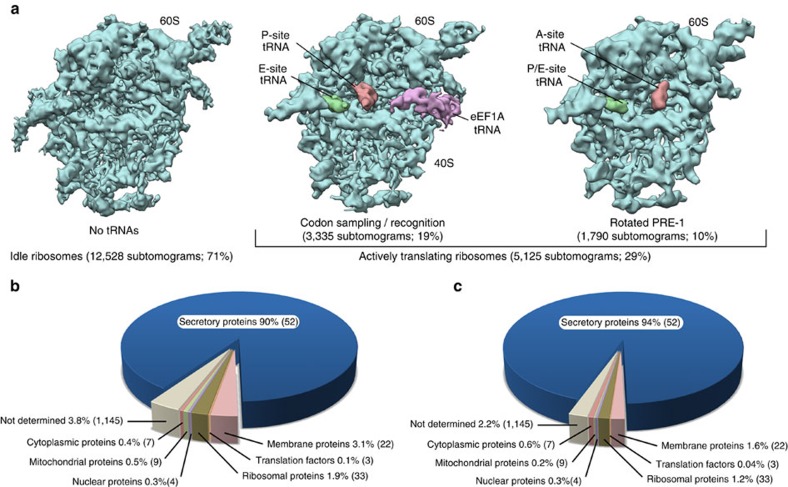
Characterization of Sec61 functional states by classification and mRNA sequencing. (**a**) Classes obtained by unsupervised classification of subtomograms focused on the tRNA binding sites. Only the 60S subunit is shown oriented such that the ER membrane corresponds to the paper plane. Resolution of the densities has been determined by Fourier cross-resolution (EMDB 5592) to 9.4 Å (idle), 11.1 Å (codon sampling/recognition) and 12.5 Å (rotated PRE-1), respectively, and densities have been filtered to their respective resolution. Class abundance is specified as absolute number of subtomograms and percentage of all particles. Translational states of classes with defined density for tRNAs have been assigned based on single particle cryo-EM maps of translating ribosomal complexes (EMD 2623 and EMD 5328). (**b**,**c**) Characterization of translational activity in the rER sample by sequencing of total mRNA (**b**) and ribosome-protected mRNA fragments (**c**). Numbers of contributing mRNAs with translational activity are provided in brackets.

The absence of visible density for tRNAs in our subtomogram average already indicates that the majority of its contributing subtomograms correspond to idle ribosome-translocon complexes not engaged in translocation or membrane integration of a nascent peptide. Indeed, unsupervised classification of subtomograms focused on the tRNA binding sites confirms the complete absence of tRNAs for the majority of subtomograms (71%). For a minority of subtomograms (29%), well-defined density for tRNAs is present (Fig. 2a), indicating actively translating ribosomes. In line with the specialization of pancreatic tissue in the synthesis of extracellular digestive enzymes, ribosome profiling reveals that 94% of these actively translating ribosomes in our rER sample are engaged in the synthesis of soluble secretory proteins (Fig. 2b,c), in particular of the digestive system. The most abundant reads were mRNA fragments from pancreatic lipases as well as proteases such as anionic trypsin and chymotrypsin C. This suggests that the subtomograms in our data set depict ribosome-Sec61 complexes almost exclusively in the two non-inserting states (either idle or translocating a soluble protein), which have been proposed to be not completely identical^8^ but highly similar, in particular at a resolution of ∼9 Å (refs 5, 6).

Fourier shell cross-resolution with a cryo-EM single particle reconstruction of the human 80S ribosome^18^ indicates that the resolutions of the subtomogram averages derived from either idle (12,500 subtomograms) or translocating (5,100 subtomograms) ribosome-Sec61 complexes are 9.4 and 10.0 Å, respectively (Supplementary Fig. 5a). The high cross-resolution of the density segments representing Sec61 in the two subtomogram averages (10.2 Å at FSC=0.5; Supplementary Fig. 5b) as well as superposition of the atomic Sec61 model described below (Supplementary Fig. 5c), suggest that the idle and translocating states of Sec61 are essentially indistinguishable at the resolution range of 9–10 Å in the native translocon. Therefore, they are referred to as one single ‘non-inserting state' from here on.

### Native Sec61 is laterally open in the non-inserting state

**Figure 3:**
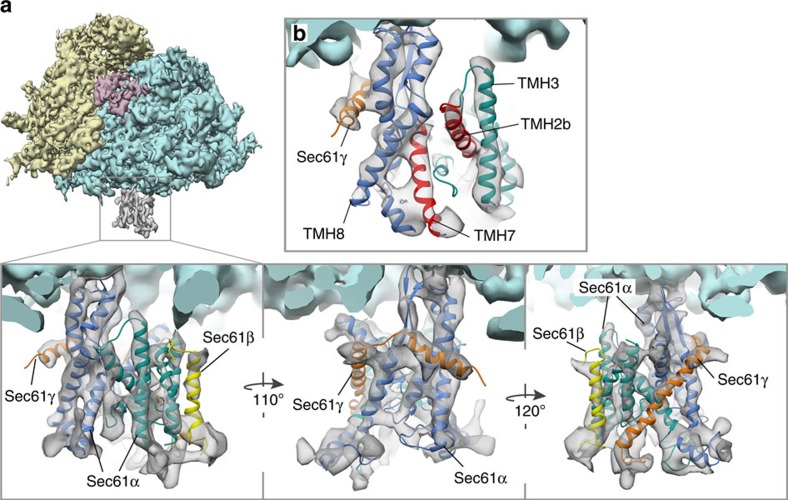
Atomic model of native Sec61 in the non-inserting state. (**a**) Isolated densities for the 40S (yellow) and 60S (light blue) ribosomal subunits, translation elongation factors (magenta) and Sec61 (grey). In the magnified views, the flexibly fitted atomic model of the heterotrimeric Sec61 complex was superposed on the density. The N- and C-terminal halves of Sec61α (green and blue, respectively), Sec61β (yellow) and Sec61γ (orange) are depicted. (**b**) View focusing on the lateral gate between TMH2b and TMH7, which are highlighted in red. Density threshold and viewing angle are different from (**a**) for optimal visualization of the lateral gate and its surroundings.

**Figure 4:**
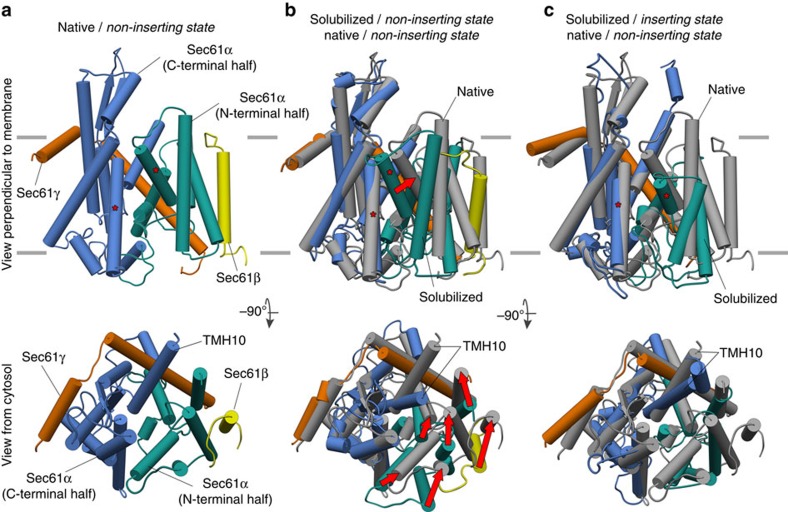
Native Sec61 adopts an open conformation in the non-inserting state. (**a**) Conformation of native Sec61 in the non-inserting state. The N- and C-terminal halves of Sec61α (green and blue, respectively), Sec61β (yellow) and Sec61γ (orange) are depicted. In the views perpendicular to the membrane the asterisks mark the two TMHs forming the lateral gate. In the views from the cytosol, TMH10 of Sec61α is annotated. (**b**) Superposition of native (grey) and solubilized Sec61 (coloured as in a; PDBs 3J7Q (ref. 8)) in the non-inserting state. Red arrows indicate the motions of TMHs in the N-terminal half of Sec61α and Sec61β linking both conformations. (**c**) Conformations of native Sec61 in the non-inserting state (grey) and solubilized SecYE mimicking the inserting state (coloured as in a; PDB 3MP7 (ref. 3)).

As classification according to tRNA occupancy revealed that the majority of particles contributing to our subtomogram average (71%) corresponds to idle ribosome-translocon complexes, we fitted an atomic model of the complete idle ribosome-Sec61 complex (PDBs 3J7Q and 4W23) into our subtomogram average as a rigid body. With this global fit, we observed excellent co-localization of rod-like densities in the translocon part of the average and α-helices in the atomic model for the C-terminal domain of Sec61α and Sec61γ (Supplementary Fig. 6a). In contrast, for the N-terminal domain of Sec61α and Sec61β, helices in the atomic model and the subtomogram average do not match. However, when the N-terminal domain of Sec61α and Sec61β are fitted as a separate rigid body, co-localization of helical densities in the average and α-helices in the atomic model is achieved also for the N-terminal domain of Sec61α and Sec61β (Supplementary Fig. 6b). The only α-helix, which was not completely positioned in a rod-like density for either of the two arrangements, is the very C-terminal TMH10 of Sec61α. A translation of this helix by ∼13 Å along its axis towards the ribosome entirely embeds it in a well-defined rod-like density (Supplementary Fig. 6c). The initial model, resulting from the two described rigid body fits and the adjustment of TMH10 served as an initial model for molecular dynamics flexible fitting (MDFF)^19^. Motions of the initial model during MDFF were small (root-mean squared deviation of C_α_ atoms <3 Å Supplementary Fig. 6d), but position helices more accurately into the density (Fig. 3, Supplementary Fig. 7, Supplementary Movie 1), revealing the conformation of native Sec61 in a non-inserting state (Fig. 4a).

Compared with the starting model, the C-terminal domain of Sec61α and consequently the interaction with the ribosome remain largely unaltered with the exception of TMH10, which moves 13 Å towards the ribosome. This considerable repositioning of TMH10 into immediate proximity to the ribosome might indicate a functional role of this helix in protein transport. In addition to serving as a ‘promiscuous guiding surface' for incoming nascent chains, as previously proposed^3^, it might also act as a ribosome sensor, which may induce conformational switching of the channel in the native translocon on binding of a ribosome. In contrast to the invariant position of the C-terminal half of Sec61α, the N-terminal domain of Sec61α, in concert with Sec61β, undergoes a rigid body movement (rotation: 22°; translation along rotation axis: 13.8 Å), resulting in an open lateral gate in the native Sec61 complex (Fig. 4b, Supplementary Movie 2). Thus, when in the context of the complete translocon, native non-inserting Sec61 clearly adopts a laterally open conformation akin to the conformation first observed in X-ray crystallographic structures of an isolated SecYEG-SecA complex^4^, and an isolated SecYE complex, which supposedly mimics the inserting state of the protein-conducting channel^3^ (Fig. 4c). Similar open conformations have also been described for detergent-solubilized Sec61/SecYEG complexes engaged in the integration of a nascent TMH or signal sequence through the lateral gate into the membrane^6^,^7^, but resolved worse than in our structure of the native translocon.

## Discussion

We have obtained a subtomogram average of the native, non-solubilized ribosome-Sec61 complex at subnanometer resolution. Functional characterization by subtomogram classification focused on the tRNA binding sites and deep sequencing of ribosome-protected mRNA fragments indicates that Sec61 is in the non-inserting state (idle or translocating a soluble protein domain). The high amount of tRNA-free ribosomes observed in our study (∼70%) is consistent with recent single particle cryo-EM studies of detergent-solubilized porcine pancreatic ribosome-Sec61 complexes^8^, where even more tRNA-free ribosomes (∼87%) were observed. Although exclusively very mild conditions were used for sample preparation throughout, we cannot entirely rule out peptidyl-tRNA hydrolysis and thus the presence of a nascent chain in some Sec61 complexes classified as ‘idle'. Consequently, our functional characterization might underestimate the proportion of Sec61 complexes engaged in translocation of a soluble protein due to the presence of nascent chains on tRNA-free ribosomes.

The discrepant conformations of native and detergent-solubilized Sec61 in the non-inserting state^6^,^7^,^8^ may be explained by the effects of detergent solubilization or by the interaction with the accessory translocon components TRAP and OST. The differing physical properties of a membrane bilayer compared with a detergent micelle and the tight complex formation with TRAP and OST as observed in the native translocon may trigger a conformational change from the ‘open' to the ‘closed' conformation on solubilization. However, the presence of a nascent transmembrane helix or signal sequence in the lateral gate seems to stabilize the open state also on solubilization of Sec61, as indicated by the open conformations observed in recent cryo-EM studies^6^,^7^.

Recent mechanistic models, which were derived from the cryo-EM maps of detergent-solubilized samples in distinct functional states, suggested that ribosome-bound Sec61 is mostly present in a closed conformation and only opens transiently for integration of a nascent transmembrane helix^6^,^7^,^8^. In contrast, based on the structure of the native ribosome-Sec61 complex in the non-inserting state, we conclude that the laterally open conformation of Sec61 may be the only major conformation present in the fully assembled ribosome-bound translocon complex independent of its functional state (idle, translocating, inserting a nascent TMH). In general, a constitutively opened lateral gate would support a model^20^ of nascent chains sliding along the interior surface of the opened lateral gate. Depending on their hydrophobicity, the nascent chains would either partition into the lipid bilayer or move across the membrane into the ER lumen. The ‘sliding model' is thought to represent the lowest-energy trajectory for membrane insertion of a nascent transmembrane helix and recapitulates well how nascent chains behave in coarse-grained molecular dynamics simulations^21^. Thus, during passage through Sec61, all nascent peptides might be exposed to hydrophobic lipid tails protruding into the Sec61 channel via the lateral gate. The laterally ‘pre-opened' conformation of Sec61 observed in our structure may consequently provide immediate access to the lipid bilayer for hydrophobic nascent polypeptide stretches emerging from the ribosome, such as TMHs or signal sequences, as previously suggested based on site-specific crosslinking^22^. A largely invariant conformation of ribosome-associated Sec61 would also support that membrane integration and folding of membrane proteins is primarily driven by the thermodynamic behaviour of the growing nascent chain^20^,^23^,^24^. The structure of non-ribosome associated Sec61 in the native membrane and its assembly state remain uncharted. Nevertheless, it is likely that Sec61 adopts its closed conformation in a ribosome-free idle state in the native system, to maintain the necessary ion permeability barrier. Thus, our structure is consistent with earlier studies mainly based on electrophysiological techniques^25^,^26^,^27^ and a recent analysis using photo-induced electron transfer^28^, which all suggested that the ribosome alone (even without a nascent chain) is sufficient for partial opening of the Sec translocon in a lipid environment. The X-ray crystallographic structures of isolated SecY in the open conformation with either SecA^4^ or a second SecY complex^3^ bound to the cytosolic loops of Sec61α, further demonstrate that ‘ligand' binding to the Sec translocon can be sufficient for lateral gate opening also in the idle state.

Our study highlights the importance of analysing the structure of membrane proteins in their native membrane environment and in complex with their physiological interaction partners and it suggests that polypeptide translocation and membrane protein insertion involve much smaller conformational changes of Sec61 than previously thought. Our results suggest that the open conformation of Sec61 is the relevant one for design and interpretation of further studies of the translocation and membrane insertion process. Thus, our structure of the translocon not only allows detailed insights into the conformation of Sec61 in the native translocon, but it also represents a solid basis for follow-up studies of both structural and biochemical nature that may results in a detailed mechanistic understanding of protein transport, membrane insertion and maturation facilitated cooperatively by Sec61 and accessory translocon components.

## Methods

### Sample preparation and CET

Rough microsomes were prepared from dog pancreas as previously described^29^, but omitting the nuclease treatment. In brief, dog pancreas was cut into small pieces and cells were homogenized using a Potter homogenizer. Rough microsomes were isolated from the homogenate using several steps of differential centrifugation and discontinuous sucrose gradient centrifugation. Microsomes were diluted to 0.25 eq μl^−1^ using ribosome buffer (20 mM Hepes, pH 7.6; 50 mM KCl; 2 mM MgCl_2_) and 3 μl were applied to lacey carbon molybdenum grids (Ted Pella, USA). After an incubation time of 60 s at 22 °C, 3 μl of 10 nm colloidal gold in ribosome buffer were added to the grid and the sample was vitrified in liquid ethane using a Vitrobot Mark IV (FEI Company, The Netherlands). Tilt series were acquired using a FEI Titan Krios transmission electron microscope equipped with a ‘K2 summit' direct electron detector (Gatan, USA), operated in movie mode with 4–7 frames per projection image (exposure time 0.8–1.4 s). The transmission electron microscope was operated at an acceleration voltage of 300 kV, a nominal defocus of 3–4 μm and an object pixel size of 2.62 Å. Single-axis tilt series were recorded from −60° to +60° (first half: −20° to +60°; second half; −22° to −60°) with an angular increment of 2° and a cumulative electron dose of 90–100 electrons per Å^2^ using the Serial EM acquisition software^30^.

### Image processing

Frames from the K2 direct electron detector were aligned using a quasi-expectation maximization protocol implemented in the MATLAB toolbox AV3 (ref. 31). Correction of phase reversals due to the contrast transfer function was performed using MATLAB scripts and PyTom^32^ on single projections by strip-based periodogram averaging^33^. Tilt series were aligned with the help of interactively located gold markers and tomograms (object pixel: 2.1 nm) were reconstructed using weighted backprojection in PyTom^32^. Template matching against a single particle cryo-EM reconstruction of the human 80S ribosome^18^ filtered to 5 nm resolution was accomplished using PyTom^32^. Different rotations of the template were sampled with an angular increment of 12.85°. Subtomograms (object pixel: 2.1 nm) centred at the coordinates of the 500 highest-scoring peaks of the cross correlation function were classified using constrained principal component analysis^34^ focusing on an area encompassing the large ribosomal subunit and the ER membrane. This classification separated ER membrane-associated ribosomes from most false-positive matches, such as gold markers, ER membrane or carbon edges. For the retained coordinates, subtomograms (110^3^ voxels, object pixel: 0.524 nm) were reconstructed individually from the weighted projections using the full tilt range (−60° to +60°). After iterative alignment using PyTom^32^, a second round of constrained principal component analysis focused on the translocon part of the subtomograms separated ribosomes bound to the OST-containing translocon from ribosomes bound to the OST-lacking translocon, remaining non membrane-bound ribosomes and false positives. For the retained coordinates, subtomograms (220^3^ voxels, object pixel: 0.262 nm) were reconstructed using only a reduced tilt range (−20° to +20°) to restrict the cumulative electron dose to 30 electrons per Å^2^. Subtomograms were iteratively aligned with PyTom, using a single particle cryo-EM reconstruction of the human 80S ribosome (EMD 5592) filtered to 2 nm resolution as an initial reference for the first iteration. The mask used for alignment encompassed the complete ribosome, Sec61 and parts of TRAP and OST. For the following iterations, the average from the previous iteration served as a reference. Angular sampling and bandpass filter for alignment were set according to the determined resolution from the previous iteration. Alignment converged after 6 iterations, as judged by resolution. During the alignment process, the resolution of the subtomogram average was determined by FSC (FSC=0.5) of two averages from each half of the data. The resolution of the converged subtomogram average was additionally assessed by cross-resolution (FSC=0.33) with a single particle reconstruction of the human 80S ribosome (EMD 5592) on appropriately masked maps. For subtomogram alignment according to the gold standard procedure, the data set was split into two independent halves and each half-set was aligned against a single particle cryo-EM reconstruction of the human 80S ribosome (EMD 5592) filtered to 2 nm resolution in the first iteration. The following iterations were performed as described above, with the difference that the two half-sets were kept independent at all time by aligning subtomograms only against their respective average. Alignment converged after eight iterations, as judged by resolution. The resolution of the two independent subtomogram averages was determined by FSC (FSC=0.143) on appropriately masked maps. Local resolution estimation was performed with BSoft using a box size of 20^3^ voxels and otherwise default settings. For visualization, the resolution-limited maps were sharpened using a B-factor of −500.

### EM-map analysis and flexible fitting

The atomic model of the idle Sec61 complex bound to the 60S ribosome (PDB 3J7Q) was fitted into the subtomogram average as a rigid body using UCSF Chimera^35^, resulting in a good initial fit for the C-terminal half of Sec61α and Sec61γ, as judged by co-localization of helices and rod-like densities. Then, the N-terminal half of Sec61α and Sec61β were fitted into the subtomogram average as a rigid body independently of the rest of the atomic model, resulting in a good initial fit for the N-terminal half of Sec61α and Sec61β. Since TMH10 of Sec61α is neither positioned accurately in the appropriate rod-like density for the rigid body fit for the C-terminal nor for the N-terminal half of Sec61α, its position was manually adjusted. The resulting atomic model of Sec61 was refined using MDFF^19^ in the Visual Molecular Dynamics software package (VMD)^36^ until convergence using a simulated annealing protocol and implicit solvent. For comparing Sec61 conformations, atomic models of Sec61 were structurally aligned according to the C-terminal half of Sec61α using UCSF Chimera.

### Ribosome profiling

Next-generation sequencing libraries of ribosome-protected fragments and total RNA were prepared using the ARTseq Ribosome Profiling Kit (epicentre) according to the manufactures protocol with the following modification. After RNase I treatment of rough microsomes 80S monosomes were isolated by sucrose gradient and protected mRNA fragments were recovered by dissociation of the monosomes into subunits. Raw single-end sequencing reads were clipped of the known adaptor sequence. Clipped reads were first mapped to *Canis lupus familiaris* rRNA, tRNA and mitochondrial non-coding RNA using Bowtie 1.0.0. Unaligned reads were then mapped to the dog genome assembly (CanFam3.1, Ensemble release 77) using GSNAP (version 2013-10-10) including annotated splice junctions. Unique aligned reads on protein coding genes were counted with HTSeq 0.6.1 and normalized for the length of the coding sequence. For total mRNA sequencing RNase I digestion was omitted.

## Additional information

**Accession codes**: EM densities have been deposited in the EMDataBank for the ribosome-Sec61 complex in the non-inserting state obtained by ‘conventional' alignment (EMD-3068) and ‘gold standard' alignment (EMD-3069), for the subsets of idle (EMD-3070) and translocating ribosome-Sec61 complexes (EMD-3071), and for a representative tomogram (EMD-3072). Atomic coordinates of native Sec61 in the non-inserting state have been deposited in the PDB with accession code 5a6u.

**How to cite this article:** Pfeffer, S. *et al*. Structure of the native Sec61 protein-conducting channel. *Nat. Commun.* 6:8403 doi: 10.1038/ncomms9403 (2015).

## Supplementary Material

Supplementary Figures Supplementary Figures 1-7

Supplementary Movie 1 Atomic model for native Sec61 in the non-inserting state. The atomic model for native Sec61 after flexible fitting (N- and C-terminal halves of Sec61α: green and blue, respectively; Sec61β: yellow; and Sec61γ: orange) was superposed on the isolated density for Sec61 and parts of the large ribosomal subunit (gray). The movie shows a view perpendicular to the ER membrane with the cytosolic face on top and the lumenal face on the bottom.

Supplementary Movie 2 Comparison of conformations for non-inserting Sec61 in the native and solubilized state. The movie shows a UCSF Chimera morph from Sec61 in the native state into Sec61 in the solubilized state and back in two cycles. The N- and C-terminal halves of Sec61α are shown in green and blue, respectively, Sec61β in yellow and Sec61γ in orange. The movie shows a view from the cytosol with the ER membrane in the paper plane.
