# Supplementary material for: Structure of the native Sec61 protein-conducting channel
Source: Nat Commun. 2015 Sep 28;6:8403. doi: 10.1038/ncomms9403 (PMC4598622; doi:10.1038/ncomms9403)
Supplement: Supplementary Figures — 1-7 [file ncomms9403-s1.pdf]

Supplementary Information

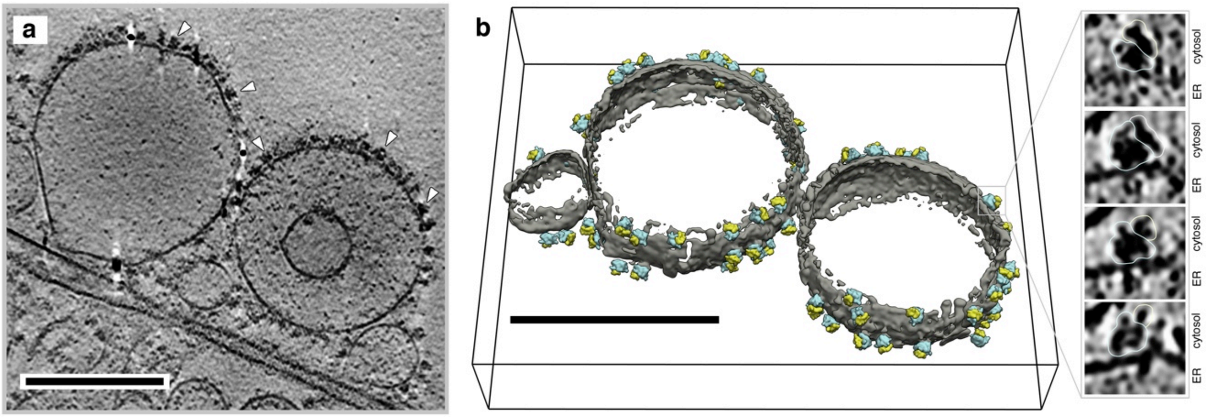

Supplementary Figure 1.Visualization of a representative tomogram depicting isolated rER vesicles. **a**, Slice through a Gaussian-filtered (1.5 nm width) tomogram depicting isolated rER vesicles. Selected ER membrane-associated ribosomes (white arrow heads) are indicated. Scale bar corresponds to 200 nm. **b,** Rendered and segmented version of the tomogram shown in (**a**). The ER membrane is depicted in gray and detected ER-membrane associated ribosomes are represented by the template (40S: yellow, 60S: blue). Scale bar corresponds to 300 nm. Right panel: Slices through a Gaussian-filtered (1.5 nm width) subvolume depicting a single ER membrane-associated ribosome. Density corresponding to the 40S and 60S subunits was annotated (yellow and blue lines delineating density, respectively).

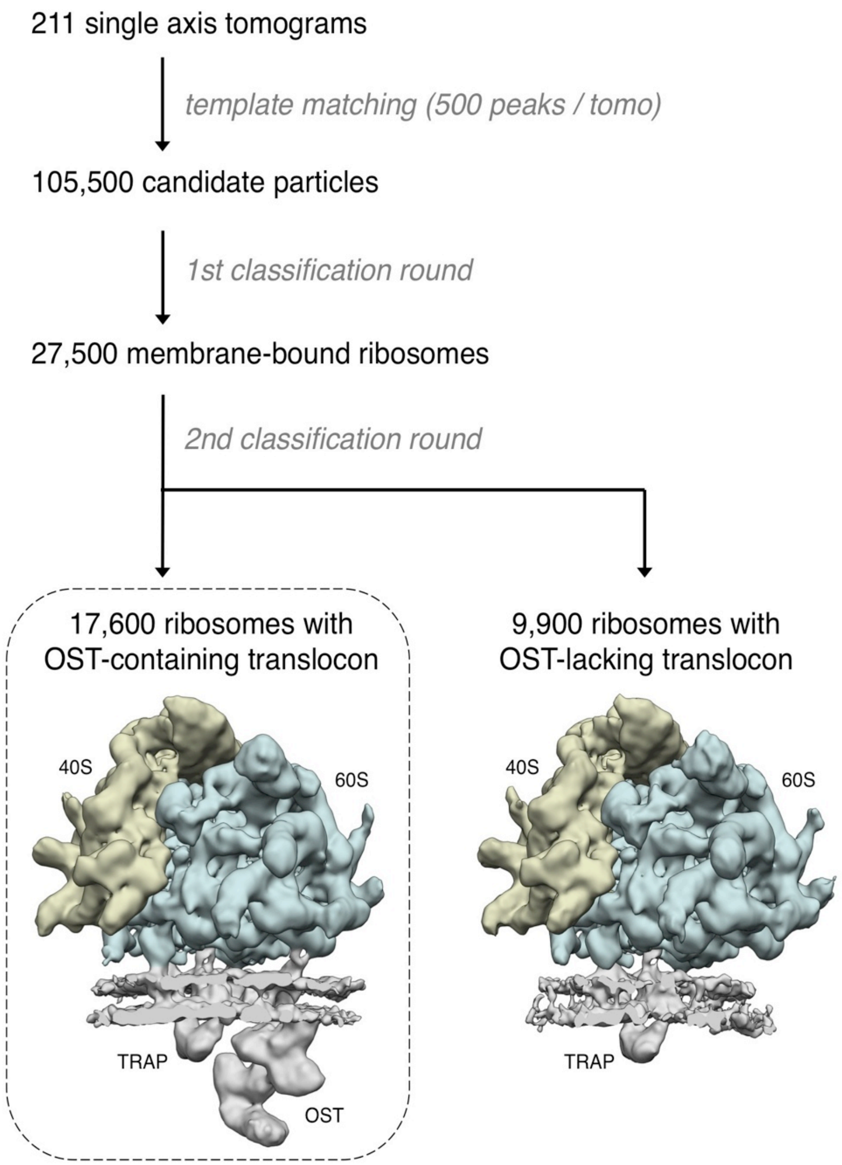

Supplementary Figure 2. Subtomogram classification workflow. From 211 single axis tomograms 105,500 candidate particles were obtained by template matching. In two consecutive rounds of classification, ribosomes bound to the OST-containing translocon (17,600 subtomograms) were separated from ribosomes bound to the OST-lacking translocon (9,900 subtomograms), not membrane-bound ribosomes and false positives.

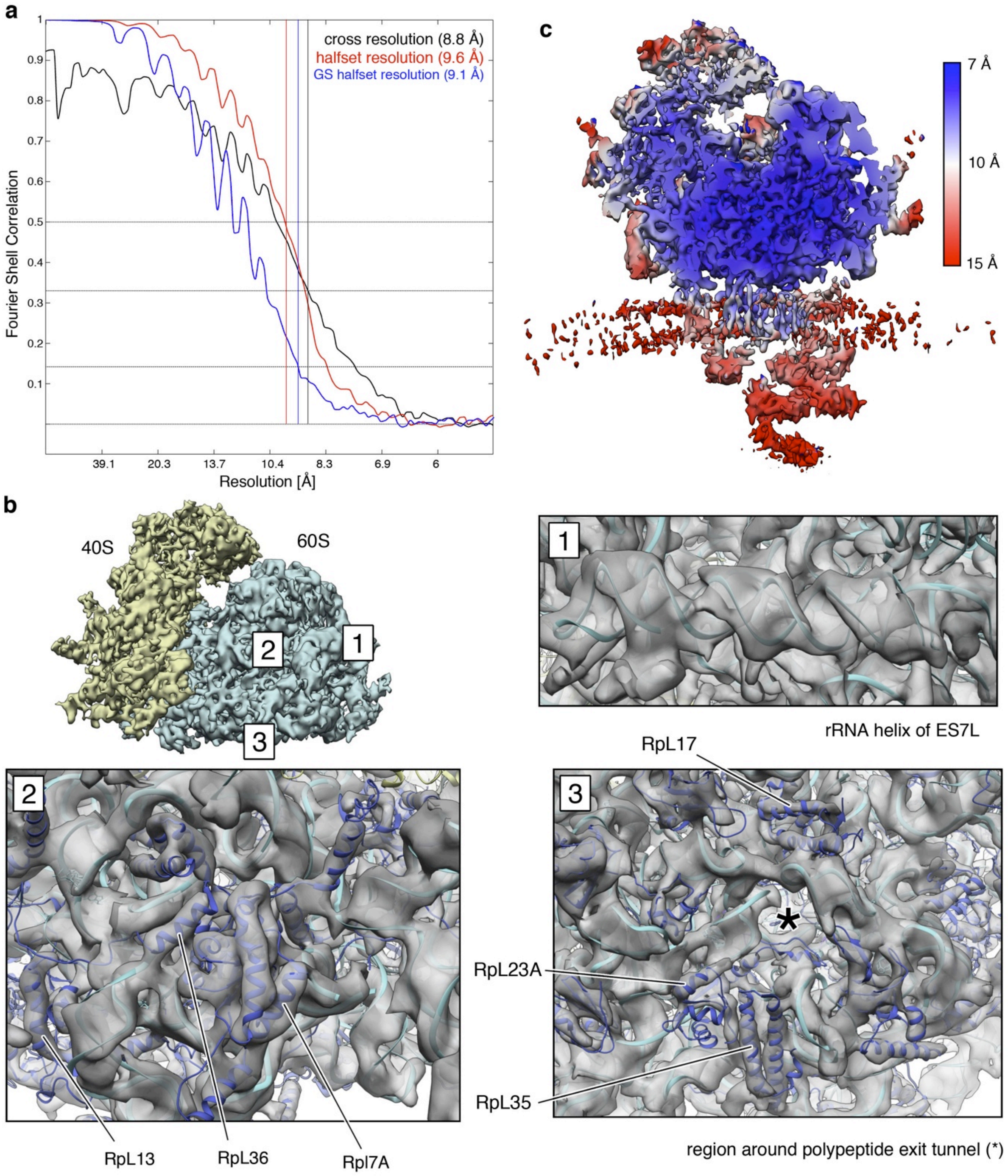

Supplementary Figure 3. Resolution assessment. a, The resolution of the ribosome part of the subtomogram average was determined to 9.6 Å by Fourier Shell Correlation (FSC 0.5 criterion) and 8.8 Å by cross-resolution (FSC 0.33 criterion) with a single particle cryo-EM map of the human 80S ribosome (EMD-5592). After subtomogram alignment following the gold standard procedure, the resolution of the ribosome was determined to 9.1 Å (FSC 0.143 criterion). **b,** Selected areas of the subtomogram average with the atomic model of the porcine

80S ribosome (PDBs 3J7Q, 4W28) superposed. Proteins and rRNA for the large subunit are depicted in dark and light blue, respectively. Illustrated areas are located on the ribosomal surface, as indicated. According to local resolution assessment (**c**) the depicted areas have a similar resolution as the density segment corresponding to Sec61. **c,** Local resolution assessment of the subtomogram average with an estimated range of 7 Å at the core of the ribosome and below 10 Å in most parts of the ribosome-associated membrane region.

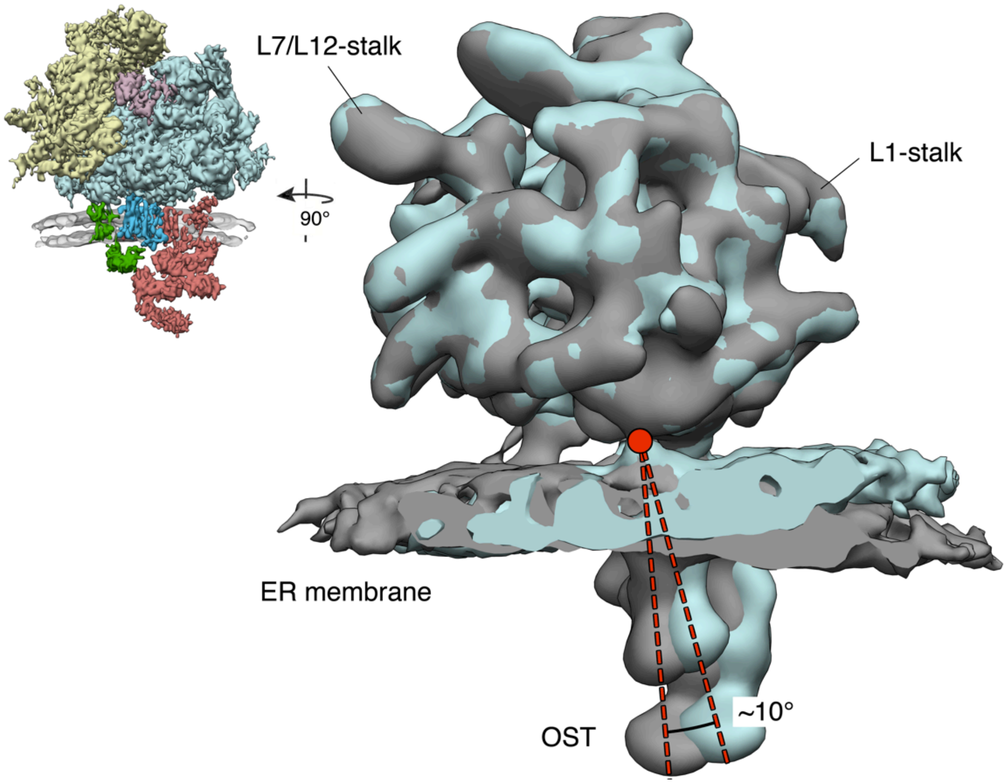

Supplementary Figure 4. Variability of ribosome-binding to the membrane. Classification of subtomograms focused on the translocon part yielded populations of ribosomes that deviate in their orientation with respect to the ER membrane and the translocon. The two class averages that deviate most from the average orientation are superposed to each other (grey, blue), visualizing the variability of ribosome-binding to the membrane. Class averages were filtered to 3 nm and oriented as indicated by the inset. The membrane was cut to allow for a better view on lumenal complexes.

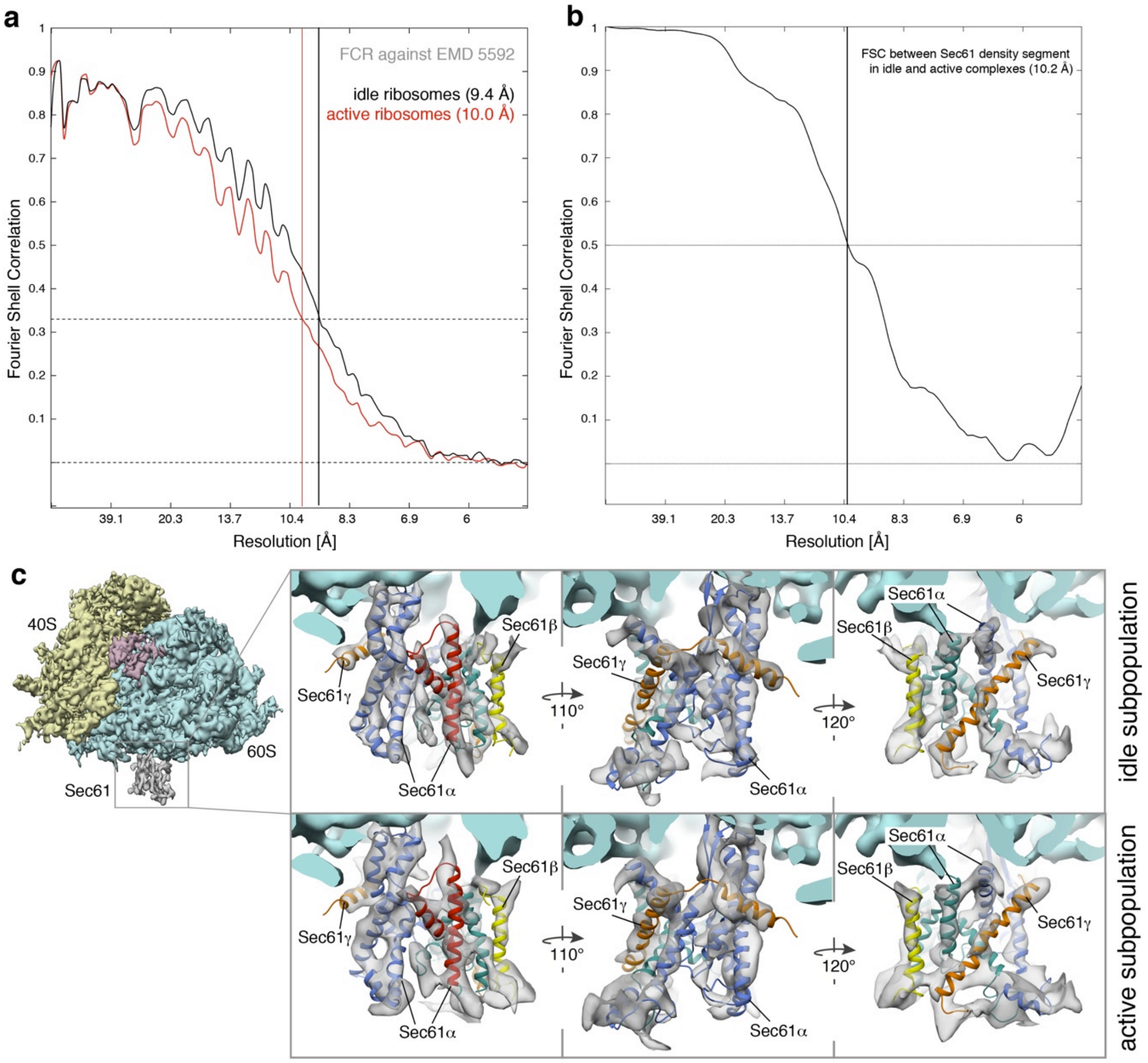

Supplementary Figure 5. The overall conformations of idle and translocating ribosome-Sec61 complexes are indistinguishable. a, For idle (black curve, 12,500 subtomograms) or translocating (red curve, 5,1000 subtomograms) ribosome-Sec61 complexes, the resolution was determined to 9.4 Å and 10.0 Å, respectively, by Fourier Shell cross-resolution (FSC 0.33 criterion) with a single particle cryo-EM map of the human 80S ribosome (EMD-5592). **b,** Fourier Shell Correlation between the density segments representing Sec61 in the idle and translocating ribosome-Sec61 complexes. **c,** Isolated densities representing idle (top) or translocating (bottom) Sec61, filtered to the determined resolutions. The model for Sec61 later obtained by flexible fitting is superposed to visualize the highly similar overall conformation of idle and translocating Sec61. TMHs 2 and 3 of Sec61α, which define the position of the N-terminal half of the lateral gate, are depicted in red.

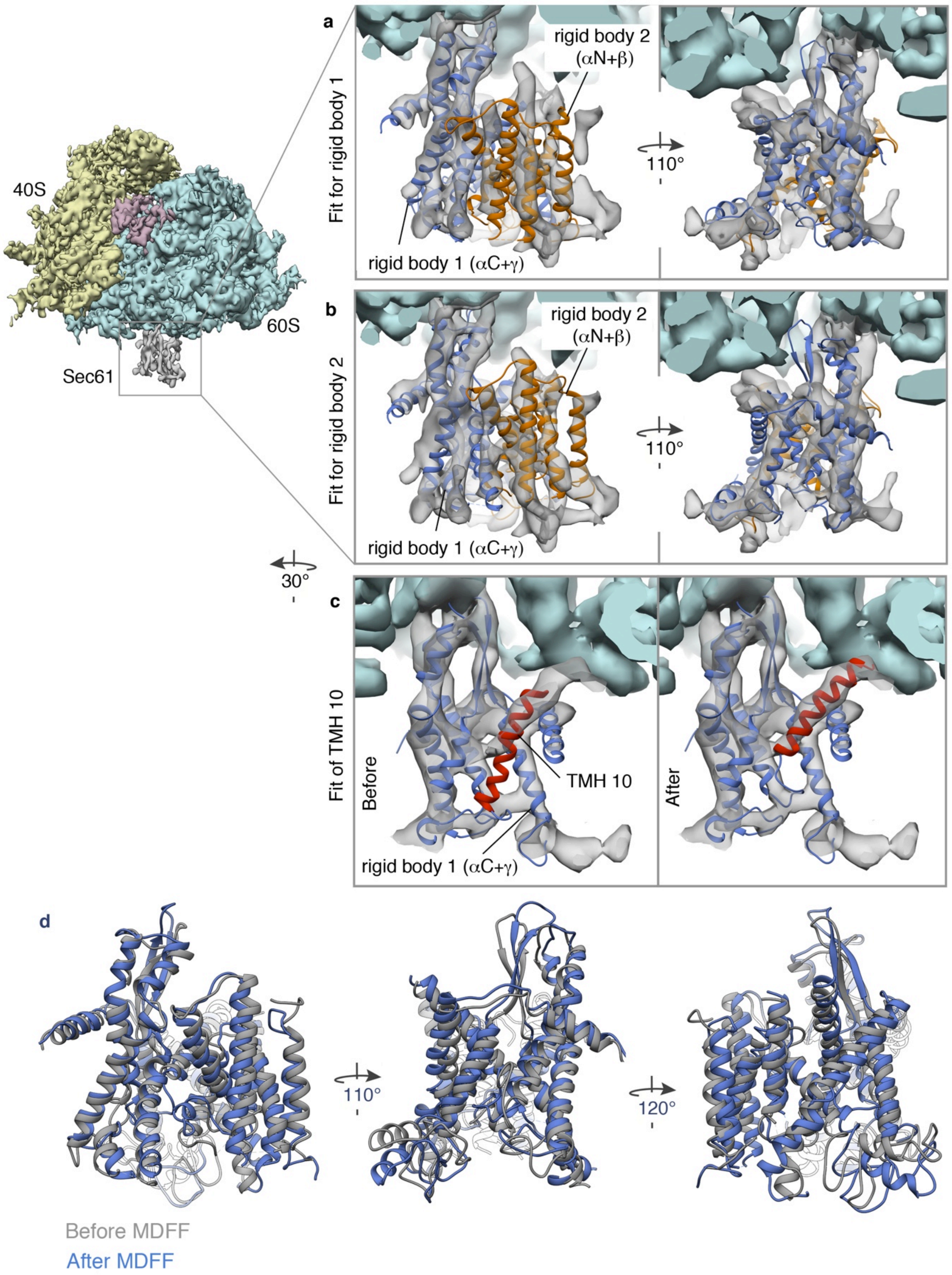

Supplementary Figure 6. Preparation of the Sec61 model used for MDFF refinement and motions during MDFF. a,b Segmented densities for the 40S (yellow) and 60S (light blue) ribosomal subunits, translation elongation factors (magenta) and Sec61 (gray). Components of the atomic model for idle Sec61 (PDB 3J7Q) were grouped into two rigid

bodies (rigid body 1/blue: C-terminal half of Sec61α + Sec61γ; rigid body 2/orange: N-terminal half of Sec61α + Sec61 β). Rigid body 1 was fitted into the density together with an atomic model of the complete 80S ribosome (PDBs 3J7Q and 4W23), while rigid body 2 was fitted independently of rigid body 1 and the ribosome to achieve co-localization between helices in the atomic model and rod-like densities. **c,** Manually guided repositioning of TMH10 (red) of Sec61α. Density and atomic model were cut to allow for a better view on TMH10. **d,** Superposition of Sec61 models before (gray) and after (blue) MDFF. Models are oriented as in Fig. 3.

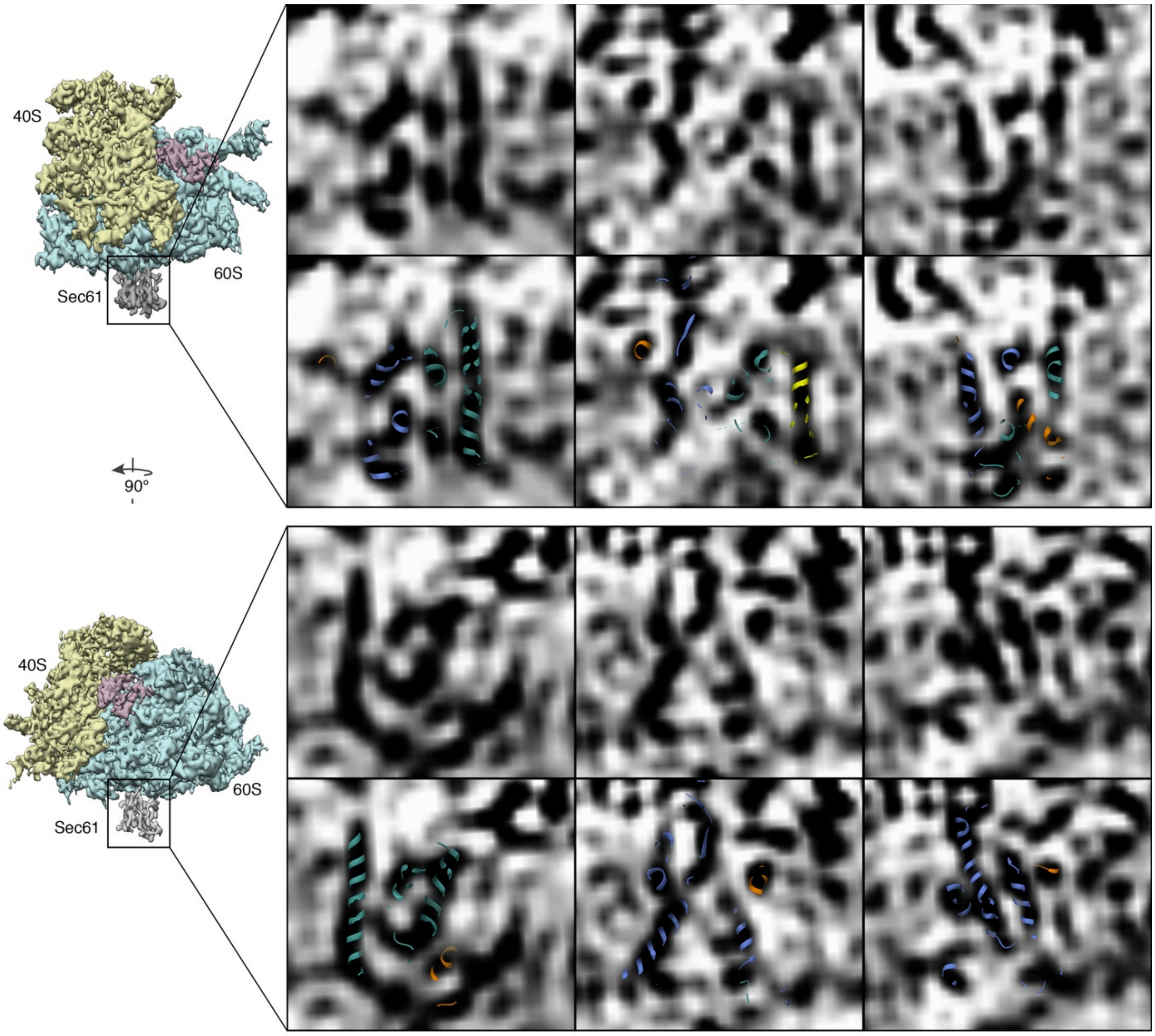

Supplementary Figure 7. Illustrative slices of the channel density. Slices of the density segment corresponding to Sec61 with (lower rows) and without (upper rows) the Sec61 model superposed. The slices are oriented as indicated by the isosurface rendered maps of the ribosome-Sec61 complex (left). Coloring corresponds to Fig. 3.
